# Morbimortality Associated with Liver Cirrhosis in Peru: An Ecological Analysis for the Period of 2004–2016

**DOI:** 10.3390/ijerph19159036

**Published:** 2022-07-25

**Authors:** Daniela Rojas-Acuña, Nilo Polo-Samillan, Angie Z. Vasquez-Chavesta, Crist Escalante-Arias, Cristhian J. Rios-Perez, Carlos J. Toro-Huamanchumo

**Affiliations:** 1 School of Medicine, Universidad Católica Santo Toribio de Mogrovejo, Chiclayo 14012, Peru; danielarojasusat@gmail.com (D.R.-A.); nilopolo68@gmail.com (N.P.-S.); angiezonaly.vc@gmail.com (A.Z.V.-C.); cristescalante23@gmail.com (C.E.-A.); cristhianrios191997@gmail.com (C.J.R.-P.); 2 Asociación Científica Médico Estudiantil USAT, Chiclayo 14012, Peru; 3 Unidad para la Generación y Síntesis de Evidencias en Salud, Universidad San Ignacio de Loyola, Lima 15024, Peru; 4 Clínica Avendaño, Unidad de Investigación Multidisciplinaria, Lima 15074, Peru

**Keywords:** liver cirrhosis, morbidity, mortality

## Abstract

Liver cirrhosis (LC) is an irreversible, chronic disease and constitutes the last clinical stage of many different liver diseases. The main cause of death is upper gastrointestinal bleeding caused by esophageal variceal rupture. We aim to depict the trend and estimate the morbimortality. For this, we conducted an ecological study and analyzed data from 2004–2016 using the public information provided by the Peruvian Ministry of Health (Ministerio de Salud del Perú, MINSA). Morbidity and mortality were presented according to 5-year groups. Regions were divided according to age quintiles for each studied year, and standardized morbidity and mortality rates were calculated for each natural geographic region; we found that LC-related morbidity per 100,000 people was 52.3 in 2004 and 117.9 in 2016. LC-related mortality had increased from 13.6 deaths per 100,000 people in 2004–2005 to 16.8 deaths per 100,000 people in 2015–2016. Morbidity showed an upward trend in Peru, especially in the departments of Callao, Ica, and Tumbes, whereas mortality showed an upward trend in the departments of Lambayeque, Ica, and Callao.

## 1. Introduction

Liver cirrhosis (LC) is a chronic, irreversible disease that constitutes the last clinical stage of different liver diseases [1]. It is characterized by an alteration of the liver architecture caused by the presence of regenerative nodules and diffuse fibrosis, leading to intrahepatic vascular alteration, portal hypertension, and consequent liver failure [2]. Worldwide, the main causes of LC are alcohol consumption, chronic hepatitis B and C infections, and nonalcoholic fatty liver disease [3].

Cirrhosis is the eleventh leading cause of death globally [4]. The World Health Organization reported that the estimated mortality caused by LC was 16.1 per 100,000 people in 2010, and it accounted for 1.9% of the global deaths [5]. However, in 2016 the mortality rate increased to 16.8 per 100,000 people, corresponding to 2.2% of the total deaths worldwide [5]. The causes of LC-related mortality are attributed to its complications, the most frequent being infections, encephalopathy, digestive hemorrhage, ascites, and hepatorenal syndrome. These require hospitalization and immediate medical attention [2]. Additionally, the main cause of death is upper gastrointestinal bleeding due to esophageal variceal rupture [3].

Eastern Europe has the highest mortality rates associated with this disease [6]. In 2016, Moldova reported a mortality rate of 79.4 per 100,000 people, making it the country with the highest LC-related mortality rate in the European continent [5]. In Latin America, in the same year, Uruguay was the country that reported the lowest mortality rate of 6.4 per 100,000 people [5]. In 2000, Peru had a mortality rate of 9.48 per 100,000 people, with LC ranking fifth among the causes of deaths and second among the causes of digestive and hepatobiliary diseases [7,8]. However, these studies were carried out based on local data alone, and no updated information is available on these data [9,10].

Some studies have been conducted worldwide to identify the prevalence or morbimortality associated with LC. However, not all of them use a standardized operationalization with the International Classification of Diseases (ICD-10), which should be used to avoid underestimating or overestimating the real figures. This is evidenced by the variety of figures presented in the published studies [11,12]. In addition, there is a lack of evidence at the national level and the need to implement and reinforce strategies to enable timely diagnosis and treatment. Therefore, the objective of the present study was to describe the trend and estimate the morbimortality associated with LC in Peru during the period of 2004–2016.

## 2. Materials and Methods

### 2.1. Study Design and Context

This was an ecological study that analyzed secondary data for the period from 2004 to 2016. The data were obtained from a public health information source of the Peruvian Ministry of Health (MINSA). The morbimortality associated with LC was analyzed in each of the 25 political regions of Peru (24 departments plus the constitutional province of Callao) distributed along its 3 natural regions: Coast, Highlands, and Jungle. The departments corresponding to the coastal region are Callao, Ica, La Libertad, Lambayeque, Lima, Moquegua, Piura, Tacna, and Tumbes; those corresponding to the Highland region are Ancash, Arequipa, Apurímac, Ayacucho, Cajamarca, Cusco, Huancavelica, Huánuco, Junín, Pasco, and Puno; and those belonging to the Jungle region are Amazonas, Loreto, Madre de Dios, San Martín, and Ucayali [13,14].

### 2.2. Data Collection and Procedure

In Peru, information regarding diagnosed diseases is maintained by the healthcare registries of the Regional Health Directorate of each department, which gathers the information issued by healthcare facilities at the national level (primary care, hospitals, and specialized institutions). Additionally, when a person dies, the healthcare facility fills out a death certificate to legalize the death of that person and establish the fundamental cause of death [15]. MINSA collects such certificates through the National Registry of Identification and Civil Status (Registro Nacional de Identificación y Estado Civil, RENIEC), under Ministerial Resolution No. 826–2005 [16]. The certificate contains a code assigned by the ICD-10 that states the underlying cause of death [17]. This ICD-10 can be specific (detailing that LC was caused by drug use) or general (LC without a specified cause) depending on the information available in each certificate.

Initially, ICD-10 K70-K77 corresponded to liver diseases. Subsequently, acute liver injury was ruled out and comparisons with ICD-10s used in other studies conducted with similar procedures were made [18,19,20,21,22,23,24]. Because of the substantial differences in the assignment of ICD-10 codes for liver-related deaths and the lack of a consistent standard definition in the published literature, we considered those ICD-10s that matched at least once in all similar studies. Hepatocellular carcinoma was excluded because despite being a liver disease, it does not depend on the presence or absence of LC. The selected codes were sent to a medical epidemiologist and two gastroenterologists for obtaining their approval. Finally, the following ICD-10s were selected: B18, I85, I86.4, I98.2, K70, K71.7, K72.1-K72.9, K73, K74, K75.2-K75.9, K76.6-K76.7, and K76.9. For the definition of morbidity, we considered all notifications of LC and/or complications associated with it (according to ICD-10). In the case of mortality, we considered all notifications in which the causes of death corresponded to any of the ICD-10 codes specified above (Table S1).

The national records of the diagnosed cases of LC and deaths caused by it were obtained from MINSA through its public information access platform. We requested the distributions of morbidity and mortality according to 5-year groups and region.

### 2.3. Variables

Morbidity: Presented as prevalence, it is the annual number of LC cases registered in the period of 2004–2016. It includes both the population with Comprehensive Health Insurance (Seguro Integral de Salud, SIS) and the unaffiliated population that attended any regional MINSA facility during the studied years.

Mortality: Annual number of deaths caused by LC recorded in the studied years. It was also compared with the all-cause mortality observed among the inhabitants of each region in each year evaluated.

Other variables of interest were age group, year, and region of the registered notification. To estimate the number of people who attended MINSA facilities during the studied years, the total population with SIS and those without health insurance were used as a proxy. This is due to the fact that a considerable percentage (>30%) of the latter group decides to go to a MINSA facility when they need medical care and pays for the care received [25]. This population estimate was obtained from the National Household Survey (Encuesta Nacional de Hogares, ENAHO) of the National Institute of Statistics and Informatics of Peru (INEI, by its acronym in Spanish).

### 2.4. Data Analysis

Relative and absolute frequencies were calculated for the data obtained and processed in a Microsoft Excel 2016 spreadsheet. Morbidity and mortality were presented according to 5-year groups. The population estimated by the World Health Organization for 2000–2025 [26] was used as reference using the direct method. Regions were divided according to age quintiles for each year of study. In addition, standardized morbidity and mortality rates were calculated for each natural geographic region according to the INEI distribution [13,14]. For both variables, the average frequencies of the first two years of study (2004–2005) and the last two years (2015–2016) were considered to avoid inaccuracies that may be caused by taking a single year as a reference.

### 2.5. Ethical Aspects

The secondary data used in this study were obtained from a public health access platform. The data are complete and exclude the personal information of individuals.

## 3. Results

**Figure 1:**
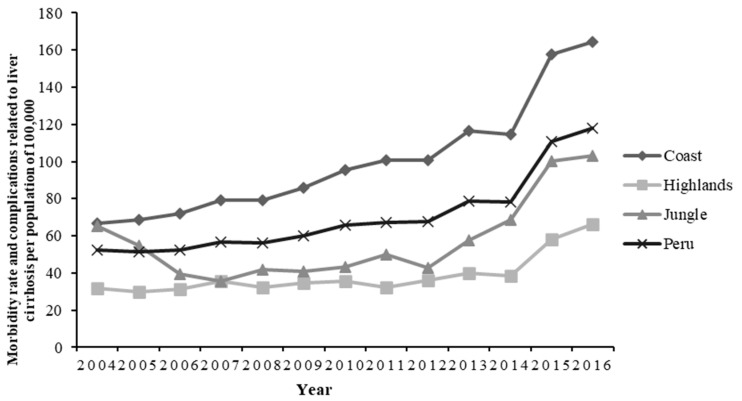
Trends of morbidity rate and complications related to liver cirrhosis according to region and registered year of death for the period 2004–2016.

In the period of 2004–2016, 199,255 cases of people suffering from LC were registered in the MINSA database. The region with the highest number of cases was the Coast (69%), and the region with the lowest number of cases was the Jungle (8.8%) (Table 1). LC-related morbidity per 100,000 people was 52.3 in 2004 and 117.9 in 2016 (Figure 1).

The overall and percentage changes were calculated for the morbidity rates obtained for the years 2004 and 2016 in the three regions. We used the next formula: {[(n2 − n1)/n1] × 100}, where n2 means morbidity rate in 2016 and n1 means morbidity rate in 2004 [27,28]. The highest increase in the number of cases was found in the Coast (147.4%), and the minimum was found in the Jungle (58%) for the studied years.

When evaluating LC-related morbidity per 100,000 people in the departments of Peru, we found that for the period of 2015–2016, the departments with the highest morbidity rates were Callao, Ica, and Lima, and the departments with the lowest mortality rates were Puno, Pasco, Huánuco, and Cajamarca (Table 2). To evaluate trends, average morbidity rates for the years 2004–2005 and 2015–2016 were calculated. We found that the morbidity increased from 52 cases of LC per 100,000 people nationally during the period of 2004–2005 to 114.4 cases of LC per 100,000 people during the period of 2015–2016. The department with the greatest decrease in LC morbidity per 100,000 people during the periods of 2005–2006 and 2014–2015 was Amazonas (−98.4%). The departments with the greatest increase in LC-related morbidity were Callao (168.8%), Ica (157.7%), and Tumbes (129.4%).

**Figure 2:**
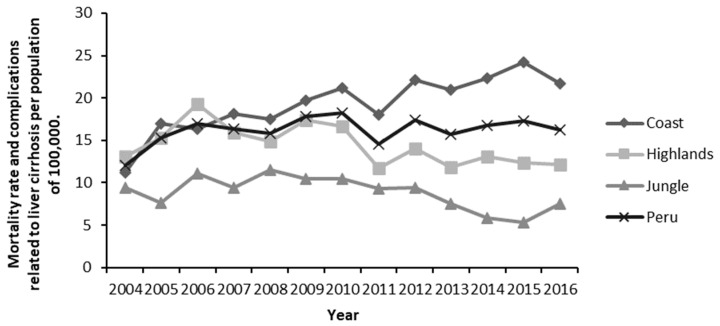
Trends of mortality rate and complications related to liver cirrhosis for the period 2004–2016 according to region and registered year of death for the period 2004–2016.

In the period of 2004–2016, 1,215,227 deaths were registered in the MINSA database; of which, 45,577 (3.8%) had LC as the underlying cause. The percentage of deaths varied from 2.9% in 2004 to 3.8% in 2016 (Table 3). Mortality due to LC per 100,000 people was 12.1 in 2004 and 16.3 in 2016 (Figure 2).

Furthermore, percentage changes were calculated for the mortality rates. An increase of 93.8% was evidenced in the number of deceased individuals in the Coast, whereas we found a 19.1% decrease in deaths in the Jungle.

When evaluating LC mortality per 100,000 people among the departments of Peru, it was found that for the period of 2015–2016, the departments with the highest mortality rates were Ica, Callao, and Lambayeque. The departments with the lowest mortality rates were Ucayali, Loreto, and Cajamarca (Table 4). To assess trends, mortality rates for the years 2004–2005 and 2015–2016 were averaged. It was found that the mortality had increased from 13.6 deaths per 100,000 people in the 2004–2005 period to 16.8 deaths per 100,000 people in the 2015–2016 period. The departments with the greatest decrease in LC-related mortality per 100,000 people between 2005–2006 and 2014–2015 were Cusco (−16.6), Apurímac (−16.2), and Ucayali (−10.1). The departments with the greatest increase in LC-related mortality were Lambayeque (14.2), Ica (13.9), and Callao (13.4) (Table 4).

## 4. Discussion

### 4.1. Main Findings

The study showed an upward trend in LC-related morbidity and mortality in Peru during the studied years (2004–2016); this upward trend was mainly observed in the coastal region. Callao, Ica, and Tumbes were the departments with the greatest increase in morbidity, whereas mortality had an upward trend in the departments of Lambayeque, Ica, and Callao.

### 4.2. Morbidity

The results showed an increase in LC-related morbidity and complications both nationally and regionally. The Coast showed the highest predominance, accounting for 69% of the cases and showing the highest percentage of change (147.4%).

The high predominance in the Coast could be explained by the fact that this area includes the most populated cities in the country (55.9% of inhabitants live in the Coast); hence, there is also more at-risk population [29]. Moreover, urban life habits, such as sedentary lifestyle, obesity, inadequate diet, and alcohol and tobacco consumption, are more frequently adopted in this area [30,31]. This increases the probability of people suffering from chronic degenerative diseases, which is evidenced by the higher prevalence of diabetes mellitus in the Coast of Peru [32]. This pathology is strongly related to LC since it can trigger it or exist simultaneously [33].

On the other hand, the increase in morbidity may be due to an increase in the disease per se or an improvement in health insurance coverage in Peru and the Peruvian Health Information System. We observed an increase (from 19.4% in 2006 to 50.2% in 2015) in the number of people who were insured with the Comprehensive Health Insurance (Seguro Integral de Salud). In addition, disease screening and access to healthcare services have improved in recent years [34]. This allows for better diagnosis and timely registration [35,36,37]. However, registration may not be comparable across regions due to disparities in the training of registration personnel as well as differences with regard to hospital equipment in each region, with the Coast being the region with the greatest coverage of healthcare [35].

We found an upward trend in Peru. This result is consistent with those of a study from Indiana [38]. A large proportion of the population of Indiana, like that of the Peruvian Coast, does not adopt healthy lifestyle habits. This is reflected in the data obtained from the Center for Disease Control and Prevention. In 2013, it was reported that 43.6% citizens consumed fruits and 26.9% consumed vegetables less than once a day. In the same year, 31.8% of the population was reported to have a body mass index >30 kg/m^2^ [39]. Moreover, Indiana ranks tenth among the worst states in USA in terms of health according to the United Health Foundation’s annual state rankings [40].

On a global burden of disease study, 40 of the 195 countries showed a decreasing trend in the age-standardized rate, with the most rapid reduction being observed in Mozambique [41]. This rapid decline may be explained by the introduction of Hepatitis B vaccination in this country [42]. LC prevalence has increased to 74.53% globally and The Caribbean is the region with the highest increase in age-standardized prevalence rate [41]. Both Latin America and the Caribbean have experienced rapid demographic and epidemiological transitions, which have resulted in important health and economic consequences. As a result, the population is not only aging rapidly but is also undergoing major lifestyle-related changes. These, in turn, have altered the disease and mortality profile of the region, resulting in a greater burden of noncommunicable diseases, such as chronic liver disease [43].

### 4.3. Mortality

An increase in mortality was detected at the national and regional levels. LC-related mortality per 100,000 people increased from 12.1 in 2004 to 16.3 in 2016. The highest percentage change was observed in the coastal region (93.8%). This differs from results in mortality between 1995 and 2000, where LC had high mortality rates in the highland, mainly in Cusco, in association with viral B hepatitis [8].

The decrease in mortality in some highland regions obtained in our study is probably due to vaccination against this virus. A study in Peruvian highland showed a decrease in LC mortality rate from 16 to 6.3 in the period of 1991–2012 after a pilot vaccination program against viral B hepatitis applied in 1991 [44]. Additionally, there should be noted that high internal migration (because of political factors, violence, terrorism, and socioeconomical conditions) occasioned high hepatitis B seroprevalence population to migrate to low endemic zones (coastal cities); therefore, epidemiologic map for viral B hepatitis may have changed in the last years thus increasing the possibility of developing LC in the Coast [11,45]. On other hand, it was shown living at high altitude is a factor that decreases the frequency of alcohol dependence [46]. This could protect highland people from LC, although further studies must be done.

Other reasons why the coast again obtained the highest percentage change are similar to those mentioned with respect to morbidity. Some of them include a higher density of population, prevalence of unhealthy habits, and prevalence of diabetes mellitus [30,31]. As it is known, this pathology can coexist with LC and/or trigger its development, thereby increasing the risk of mortality [33].

In a Chilean study, a mortality rate close to the same measure in Peru was obtained (16.6 deaths per 100,000 people) for the period 1990–2007. However, this study reported a downward trend throughout the studied years [24]. It could be explained by Chilean improvement in LC treatment and its related complications. During this time, Chile implemented ligation and sclerotherapy of esophageal varices, advances in their pharmacotherapy, portosystemic bypass through transjugular intrahepatic stent (TIPS), and liver transplantation. Moreover, Chile made an improvement in diagnosis, management, and prevention of infections in patients with LC [24,47,48].

In addition, our results differed from those of the Indiana study. Although its number of cases increased, unlike our results, its mortality rate decreased from 11.9 in 2004 to 10.0 in 2014 [38]. This was consistent with an England study, where mortality rates decreased by 2.5% during 1998–2009 [18]. In 2005, Indiana implemented a strategy for eliminating Hepatitis B virus transmission through universal childhood vaccination and improved primary prevention activities to reduce Hepatitis C virus infection [49]. In addition, Indiana alcohol consumption crude prevalence decreased from 52.5% in 2002 to 47.2% in 2010 [50]. Additionally, binge drinking among students decreased almost 10% from 2003 to 2011. Both aspects may have influenced the decrease in mortality [51,52].

Regarding England, researchers affirm mortality rates underestimated the real incidence of LC by at least three-fold between 1998 and 2009 and varied with differing definitions of disease [18]. Hence, there should be further studies taken to compare adequately. Moreover, there was an economic recession that could possibly have affected the alcohol consumption during this period [53].

A systematic analysis of LC mortality in 187 countries between 1980 and 2010, reported that LC global deaths were approximately 1.95% [21]. This finding is a result of the constant increase of the disease in the last 30 years, exceeding one million deaths in 2010. However, this study paradoxically shows a global 22% decrease in the age-standardized mortality rate. This is probably because the population size and aging rate far outweigh the overall increase in LC-related mortality [21]. In Andean Latin America, the percentage change in LC- related mortality was 0.5% during 1990–2010 period. Countries as Ecuador (−6.9%) and Bolivia (−16.5%) had results that differed considerably from Peru (+12.9%). This might be explained by a higher prevalence of metabolic syndrome in the Peruvian population at that time [54], in addition to the management of LC in Ecuador and the Bolivian strategies adopted against viral hepatitis since 2000, reducing in this way, the number of LC cases in the following years [55,56].

### 4.4. Public Health Relevance

Peru lacks strong programs focused on the education and prevention of LC and its related complications [11]. Our findings show the need to delve deeper into the specific causes of why the Coast is the most affected area. The high prevalence and increase in cases over time should encourage more education about healthy lifestyle as an effective prevention and treatment measure against LC [57]. This can be done by the population itself (self-education) or government intervention through educational programs since school [58].

Findings of our study reveal treatment and management of liver cirrhosis in Peru must be improved. Liver care in hospitals could follow treatment strategies used in other countries to avoid progression of the disease. For example, in United Kingdom, liver care is accredited at two levels: liver units (acute district hospital centers) and specialized regional centers, thereby allowing bidirectional transfer of patients between these two levels of care according to the clinical need [59].

Moreover, health centers should evaluate implementing accurate tests to the actual healthcare strategies in people with LC risk factors, thus, making possible an early diagnosis and avoiding the increase of related complications and morbidity rates. This could be affordable by further research about the most effective screening test methods for the Peruvian population considering the national budget.

Finally, it is important to review the treatments given to LC patients and their effectiveness to know if those are suitable or could be changed for more effective ones that extend their life years.

### 4.5. Strengths and Limitations

This study analyzes, with a nationally representative sample, the evolution of LC morbimortality in the Peruvian population. Previous studies only included information from the Lima region and do not have an ecological design [9,11,60].

However, some limitations should be mentioned. First, this is a secondary analysis of the information obtained from a public health information system of MINSA. Therefore, variables such as level of education or treatment received could not be addressed. Second, MINSA did not provide data on nonalcoholic LC; therefore, it was not possible to classify the evolution of LC into “alcoholic” or “nonalcoholic” LC. This classification would have allowed us to compare whether there is a different percentage of change between the two pathologies. Nevertheless, this does not affect our results because all the LC registered cases were included in the study, whether they were alcoholic or non-alcoholic. Third, there may be bias in the recorded data. This depends on the training provided to healthcare personnel in filling out the patient care and cause of death reports. For this reason, the reporting of cases or deaths may not be optimal, may have changed over the years, or differ between regions [61,62]. However, the search for data was optimized using the ICD-10 and covering the main diseases. This was achieved through a validation process conducted in consultation with specialists and based on similarities among previous studies and search patterns [18,20]. This is the first national study carried out under this criterion. Fourth, this study was limited to the MINSA population. However, it was carried out with data that closely resembles the national reality since the coverage of care offered by MINSA represents 50.2% of the entire population, a value considerably higher than that of other healthcare institutions in the country [32]. Future studies could include statistics from the private practice and social health insurance (EsSalud).

## 5. Conclusions

Morbidity and mortality caused by liver cirrhosis showed an upward trend in Peru during the period of 2004–2016. Morbidity showed an upward trend in the departments of Callao, Ica, and Tumbes, whereas a mortality upward trend was seen in the departments of Lambayeque, Ica, and Callao.

## Figures and Tables

**Table 1:** Morbidity and complications related to liver cirrhosis according to region and year of death for the period 2004-2016.

Morbidity by Natural Region							
Year	Coast		Highlands		Jungle		Total Number of Cases Related to Liver Cirrhosis in Peru
	Number of Cases	% *	Number of Cases	% *	Number of Cases	% *	Number of Cases
2004	7007	62.8	2725	24.4	1420	12.7	11,152
2005	7204	65.5	2573	23.4	1225	11.1	11,002
2006	7728	68.0	2731	24.0	903	7.9	11,362
2007	8215	67.9	3069	25.4	822	6.8	12,106
2008	8323	68.8	2816	23.3	964	8.0	12,103
2009	8979	69.3	3004	23.2	956	7.4	12,939
2010	10,118	71.1	3107	21.8	1005	7.1	14,230
2011	10,517	72.6	2796	19.3	1170	8.1	14,483
2012	10,207	71.0	3177	22.1	992	6.9	14,376
2013	12,173	71.3	3524	20.6	1385	8.1	17,082
2014	12,039	70.1	3440	20.0	1687	9.8	17,166
2015	16,962	68.6	5277	21.3	2482	10.0	24,721
2016	17,926	67.6	6008	22.6	2599	9.8	26,533
TOTAL	137,398	69.0	44,247	22.2	17,610	8.8	199,255

* Percentage is given by dividing the number of cases per region and the total number of cases per year.

**Table 2:** Morbidity and complications of liver cirrhosis by region: Comparing periods of 2004–2005 and 2015–2016.

Region	Morbidity Rate per Population of 100,000			
	2004–2005 (T1)	2015–2016 (T2)	Differences in Morbidity Rate (T2-T1)	Ratio of Morbidity Rates [T2/T1]
*Peru (Country)*	52.0	114.4	62.4	2.2
*Coastal Region*	58.3	153.0	94.7	2.6
Callao	130.2	299.0	168.8	2.3
Ica	54.6	212.3	157.7	3.9
La Libertad	66.2	74.5	8.3	1.1
Lambayeque	23.3	124.2	100.8	5.3
Lima	82.7	193.8	111.1	2.3
Moquegua	57.1	96.7	39.6	1.7
Piura	27.9	81.2	53.3	2.9
Tacna	63.3	146.4	83.0	2.3
Tumbes	19.1	148.6	129.4	7.8
*Highlands Region*	30.6	61.4	30.8	2.0
Ancash	30.3	48.6	18.3	1.6
Apurímac	32.3	70.4	38.1	2.2
Arequipa	67.9	154.5	86.6	2.3
Ayacucho	38.3	68.2	29.9	1.8
Cajamarca	21.4	35.5	14.1	1.7
Cusco	57.0	104.8	47.9	1.8
Huancavelica	14.6	41.7	27.1	2.9
Huánuco	17.0	34.9	17.9	2.1
Junín	21.8	74.4	52.6	3.4
Pasco	23.3	27.1	3.8	1.2
Puno	13.4	15.8	2.4	1.2
*Jungle Region*	63.3	110.3	46.9	1.7
Amazonas	136.8	38.4	−98.4	0.3
Loreto	60.4	76.5	16.1	1.3
Madre de Dios	54.2	165.7	111.5	3.1
San Martin	33.3	137.6	104.3	4.1
Ucayali	31.8	133.1	101.2	4.2

**Table 3:** Mortality and complications related to liver cirrhosis according to region and the registered year of death for the period 2004–2016.

Mortality by Region									
Year	Coast		Highlands		Jungle		Total Number of Deaths Related to Liver Cirrhosis in Peru		Total Number of Overall Registered Deaths
	Number of Deaths	% *	Number of Deaths	% *	Number of Deaths	% *	Number of Deaths	% ^†^	Number of Deaths
2004	1176	2.4	1126	3.4	204	4.1	2506	2.9	87,189
2005	1777	3.5	1324	4.0	172	3.5	3273	3.7	88,704
2006	1632	3.8	1547	4.4	232	4.8	3411	4.1	82,620
2007	1883	3.8	1378	4.1	216	4.6	3477	4.0	87,496
2008	1729	3.3	1231	3.7	253	4.7	3212	3.5	91,290
2009	2063	3.8	1511	4.3	246	4.4	3820	4.0	95,722
2010	2242	3.8	1453	4.3	243	4.6	3936	4.0	99,334
2011	1878	3.2	1025	3.2	219	4.0	3646	3.8	96,852
2012	2242	3.8	1233	3.7	219	4.3	3694	3.8	97,951
2013	2185	3.6	1050	3.2	182	4.0	3417	3.5	98,616
2014	2351	3.8	1173	3.8	145	3.7	3669	3.8	96,460
2015	2601	4.3	1120	3.7	133	3.3	3854	4.0	95,752
2016	2364	3.9	1107	3.5	191	3.5	3662	3.8	97,241
TOTAL	26,123	3.6	16,278	3.8	2655	4.1	45,577	3.8	1,215,227

* Percentage is given by dividing the number of deaths by the total of deaths per region/year. ^†^ Percentage is given by dividing the number of deaths by the total number of deaths per year.

**Table 4:** Mortality and complications related to liver cirrhosis by region: Comparing periods of 2004–2005 and 2015–2016.

Region	Mortality Rate per Population of 100,000			
	2004–2005 (T1)	2015–2016 (T2)	Rate Difference (T2-T1)	Rate Ratio [T2/T1]
*Peru (Country)*	13.6	16.8	3.2	1.2
*Coast Region*	14.3	23.1	8.9	1.6
Callao	16.3	29.8	13.4	1.8
Ica	16.4	30.3	13.9	1.8
La Libertad	14.5	25.7	11.1	1.8
Lambayeque	12.7	27.0	14.2	2.1
Lima	13.6	20.5	6.9	1.5
Moquegua	12.0	19.1	7.1	1.6
Piura	15.2	24.9	9.7	1.6
Tacna	14.9	22.7	7.9	1.5
Tumbes	12.8	8.3	−4.5	0.6
*Highlands Region*	14.1	12.1	−1.9	0.9
Ancash	9.5	12.3	2.8	1.3
Apurímac	23.4	7.2	−16.2	0.3
Arequipa	17.9	22.1	4.2	1.2
Ayacucho	11.9	8.9	−3.1	0.7
Cajamarca	6.6	6.1	−0.5	0.9
Cusco	29.4	12.9	−16.6	0.4
Huancavelica	10.3	10.6	0.3	1.0
Huánuco	10.3	11.5	1.2	1.1
Junín	14.6	17.1	2.5	1.2
Pasco	7.7	12.9	5.2	1.7
Puno	13.2	11.9	−1.3	0.9
*Jungle Region*	8.2	7.2	−1.0	0.9
Amazonas	7.9	7.8	−0.1	1.0
Loreto	5.6	4.1	−1.5	0.7
Madre de Dios	3.6	10.9	7.3	3.0
San Martin	10.2	9.8	−0.4	1.0
Ucayali	13.5	3.4	−10.1	0.3

## Data Availability

The data were obtained from a public health information source of the Peruvian Ministry of Health (MINSA), which is freely accessible.

## Supplementary Materials

The following supporting information can be downloaded at: https://www.mdpi.com/article/10.3390/ijerph19159036/s1, Table S1: ICD-10 codes used in this study.
