# Supplementary material for: Morbimortality Associated with Liver Cirrhosis in Peru: An Ecological Analysis for the Period of 2004–2016
Source: Int J Environ Res Public Health. 2022 Jul 25;19(15):9036. doi: 10.3390/ijerph19159036 (PMC9332628; doi:10.3390/ijerph19159036)
Supplement: Supplementary file 1 [file ijerph-19-09036-s001.zip › ijerph-1741825-supplementary.pdf]

Table S1. ICD-10 codes used in this study.

ICD Code	ICD-10 Description
B18	Chronic viral hepatitis
I85	Esophageal varices
I86.4	Gastric varices
I98.2	Oesophageal varices without bleeding in diseases classified elsewhere
K70	Alcoholic liver disease
K71.7	Toxic liver disease with fibrosis and cirrhosis of liver
K72.1	Chronic hepatic failure
K72.9	Hepatic failure, unspecified
K73	Chronic hepatitis, not elsewhere classified
K74	Fibrosis and cirrhosis of liver
K75.2	Nonspecific reactive hepatitis
K75.3	Granulomatous hepatitis, not elsewhere classified
K75.4	Autoimmune hepatitis
K75.8	Other specified inflammatory liver diseases
K75.9	Inflammatory liver disease, unspecified
K76.6	Portal hypertension
K76.7	Hepatorenal syndrome
K76.9	Liver disease, unspecified
